# System analysis of the sequencing quality of human whole exome samples on BGI NGS platform

**DOI:** 10.1038/s41598-021-04526-8

**Published:** 2022-01-12

**Authors:** Vera Belova, Anna Pavlova, Robert Afasizhev, Viktoriya Moskalenko, Margarita Korzhanova, Andrey Krivoy, Valery Cheranev, Boris Nikashin, Irina Bulusheva, Denis Rebrikov, Dmitriy Korostin

**Affiliations:** Center for Precision Genome Editing and Genetic Technologies for Biomedicine, Pirogov Medical University, Ostovityanova str. 1, Moscow, Russian Federation 117997

**Keywords:** Genomics, Sequencing

## Abstract

Human exome sequencing is a classical method used in most medical genetic applications. The leaders in the field are the manufacturers of enrichment kits based on hybridization of cRNA or cDNA biotinylated probes specific for a genomic region of interest. Recently, the platforms manufactured by the Chinese company MGI Tech have become widespread in Europe and Asia. The reliability and quality of the obtained data are already beyond any doubt. However, only a few kits compatible with these sequencers can be used for such specific tasks as exome sequencing. We developed our own solution for library pre-capture pooling and exome enrichment with Agilent probes. In this work, using a set of the standard benchmark samples from the Platinum Genome collection, we demonstrate that the qualitative and quantitative parameters of our protocol which we called “RSMU_exome” exceed those of the MGI Tech kit. Our protocol allows for identifying more SNV and indels, generates fewer PCR duplicates, enables pooling of more samples in a single enrichment procedure, and requires less raw data to obtain results comparable with the MGI Tech's protocol. The cost of our protocol is also lower than that of MGI Tech's solution.

## Introduction

Human exome sequencing is the most important method for studying hereditary pathologies today as it allows for both diagnostics and research. At the current level of research and technical development, exome sequencing has a number of benefits when compared to genome sequencing in research and clinical diagnostics. Exome sequencing allows focus on the study of the most clinically valuable genomic regions represented by protein encoding sequences. Exons and intronic splicing sites harbour approximately 85% of genetic variants responsible for hereditary diseases in humans^1^.

Implementing genomic technologies into clinical practice is significantly affected by economic factors. Unlike genome sequencing which requires reading of approximately 3 billion base pairs (bp) of the human genome, exome sequencing requires capturing and target reading of coding and adjacent regions that account for 1–2% of the human genome. On average, over the last decade, performing exome sequencing is 4–5 times cheaper per patient than performing genome sequencing^2^. At the same time, the efficiency of genome sequencing in diagnostics is only 1–2% higher than that of exome sequencing, as only a fraction of the registered in ClinVar pathogenic variants cannot be detected by the known exome kits^3,4^.

Most solutions for exome enrichment are designed for the Illumina sequencers. The most known kits include SureSelect (Agilent), SeqCap EZ (Roche NimbleGen), TruSeq Capture (Illumina). The principle of this method lies in the hybridization of biotinylated DNA or RNA probes with the complementary exome fragments from DNA libraries. Generally, enrichment kits differ in the size of target regions, probe length, its type and density, as well as the number of samples enriched in the same reaction^5^. As manufacturers strive to improve their protocols each year, new studies comparing the kits emerge^6–10^. The following parameters are compared in the first place: target enrichment efficiency, coverage uniformity, sequencing complexity, and the ability to call true single nucleotide variants (SNVs) and small insertions and deletions (indels). Currently, Illumina is a major next generation sequencing (NGS) platform which produces nearly 90% of sequencing data^11^. At the end of 2017, Chinese company MGI Tech presented the MGISEQ-2000 (now DNBSEQ-G400) as a platform for large and medium scale genome sequencing. The specific features of the MGISEQ platform are combinatorial probe-anchor synthesis (cPAS) sequencing technology and nanoballs (DNB) generated from circular molecules of DNA library by rolling circle replication^12^. There were a few studies on MGISEQ-2000 performance over the past two years, with most of them concluding that the sequencing quality of this platform is comparable to Illumina^13–16^.

MGISEQ-2000 is compatible with two commercial kits for exome enrichment, proprietary products MGI Tech MGIEasy Exome Capture V4 Probe Set and MGIEasy Exome Capture V5 Probe Set. The only difference between them lies in different probe versions, while they share the same enrichment protocol. We tested their kit MGIEasy Exome Capture V4 Probe Set produced in 2019 using the reference sample NA12891 from the Platinum Genomes project, which is a benchmark for quality analysis of various genomic protocols^17^.

**Figure 1:**
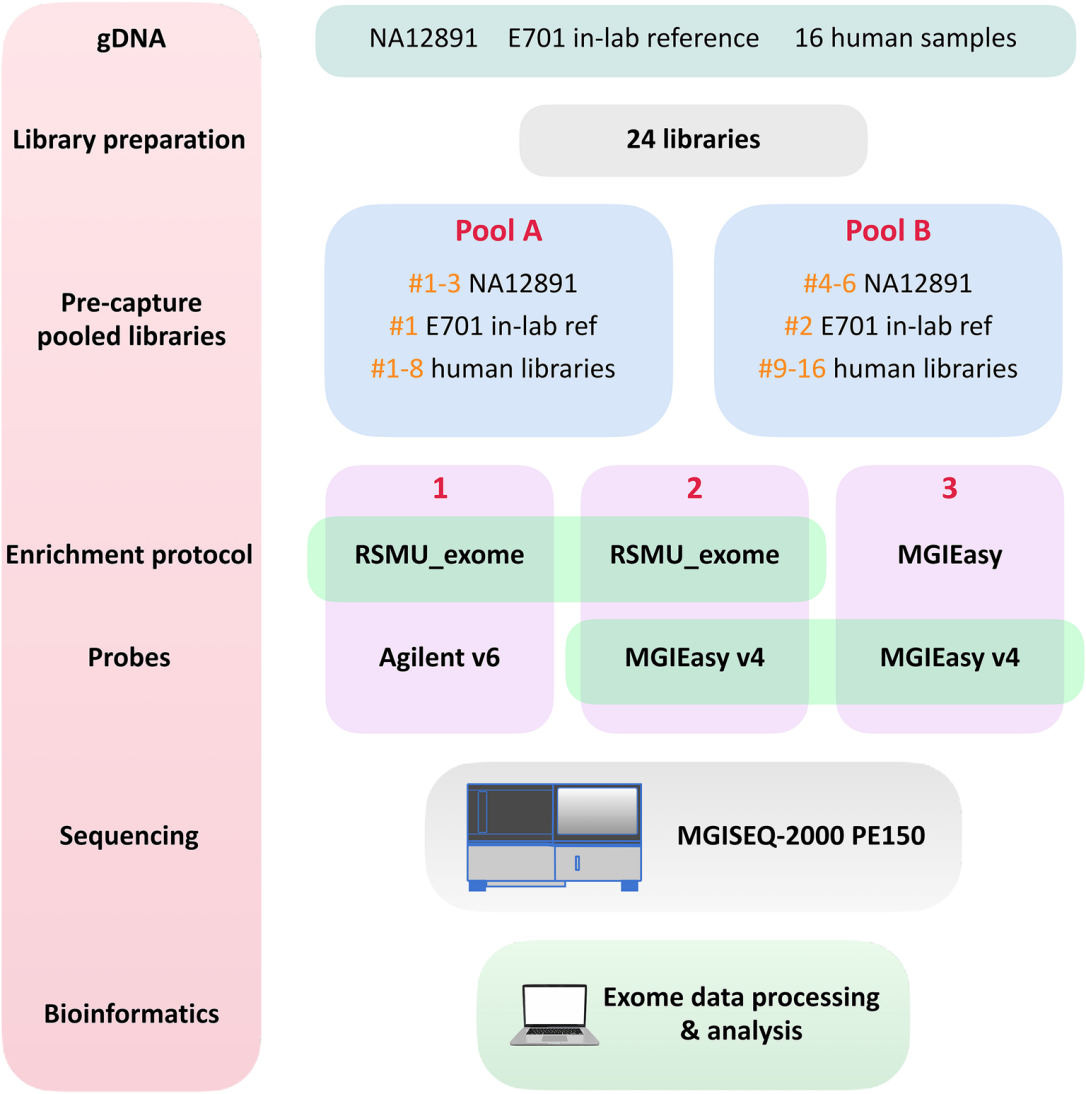
Experiment scheme. For our experiment, we used a collection of gDNA samples: NA12891 (part of the Platinum Genome project)^17^, E701 (our in-lab reference sample), and 16 human gDNA samples. We prepared 24 libraries: six libraries from gDNA NA12891, two libraries from E701, and one library each from samples 1–16. We designed two pools (**A**, **B**) with 12 libraries each. Each pool contained three libraries from NA12891 gDNA, one library from in-lab reference E701, and eight libraries from human genomic DNA samples. For protocol comparison, each of the pools was enriched using one of the enrichment protocols, our RSMU_exome using two probe options: Agilent all-exon v6 or MGIEasy V4 Probe Set, and original MGIEasy Exome protocol using MGIEasy V4 Probe Set. Note that for the MGIEasy protocol + MGIEasy V4 Probe Set variant, the number of libraries in the pool was reduced to eight according to the manufacturer's protocol. Therefore, we sequenced six independently enriched pools and obtained the dataset comprising 64 pairs of fastq files. We then performed bioinformatic and statistical analysis of the obtained data.

In the absence of a large selection of kits and protocols for the brand-new MGISEQ-2000 (DNBSEQ-G400) sequencer, we present our improved hybridization and capture method for whole exome sequencing (WES). We tested the performance of our custom protocol “RSMU_exome” and its compatibility with the MGIEasy v4 probes and the Agilent SureSelect All Exon v6 probes (hereinafter MGIEasy v4 and Agilent v6) (Fig. 1). We prepared 24 human gDNA libraries and divided them into pools A and B of 12 each. Six of these 24 libraries were prepared from gDNA of reference sample NA12891 of the Platinum Genome project^17^. To compare the protocols, each pool A and B were enriched following three different protocols: our protocol “RSMU_exome” with the Agilent v6 probes or with the MGIEasy v4 probes and the standard protocol MGIEasy Exome Capture V4 Probe Set. This experiment resulted in six differently enriched pools of libraries which we sequenced and then compared pairwise for exome quality bioinformatics parameters.

## Material and methods

### Ethics statement

This study conformed to the principles of the Declaration of Helsinki. The appropriate institutional review board approval for this study was obtained from the Ethics Committee at the Pirogov Medical University. All patients provided written informed consent for sample collection, subsequent analysis, and publication thereof.

### Sample collection

In this study, we used the reference DNA sample NA12891. We isolated DNA from the blood samples taken from 16 patients and from our in-laboratory patient reference blood sample.

### DNA extraction

DNA isolation was performed using the DNeasy Blood and Tissue Kit (Qiagen) according to the manufacturer's protocol. The extracted DNA was quantified with the Qubit dsDNA BR Assay system (Life Technologies) and its quality was assessed by 1% agarose gel electrophoresis.

### Library preparation

We prepared one library for each DNA sample from 16 patients, two independent libraries for our in-lab reference sample E701, and six independent libraries for the NA12891 sample (in sum 24 libraries). For each library, 400 ng of input genomic DNA was sheared to ~ 300 bp fragments with the Covaris E220 System according to the recommended manufacturer’s procedure. The size-selection of DNA fragments was performed using the SPRI AMPure XP beads (Beckman Coulter) to achieve a target peak of 240 to 290 bp (the first cut ratio of × 0,8, the second cut ratio of × 0,2).

Apart from the PCR step described below, all further steps of library preparation were performed using the MGIEasy Universal DNA Library Prep Set (MGITech). For the further pooling of individual libraries and elimination of hopped reads, balanced combinations of barcoded adapters from the MGIEasy DNA Adapters-96 (Plate) Kit were selected using the BC-store software developed in our laboratory^18^ according to the strict criteria. Therefore, each library was ligated to an adapter containing a single selected barcode. To amplify the libraries, we used KAPA Hi-Fi polymerase (KAPA Biosystems) instead of MGI polymerase according to the KAPA manufacturer's recommendations, with the following PCR program: a 3-min activation step at 95 °C, 10 cycles consisting of three steps (20 s at 98 °C, 15 s at 60 °C, 30 s at 72 °C), and the final 10-min extension step at 72 °C. When needed we added two cycles of PCR for reach the required DNA amount. Quality control of the DNA libraries was performed by gel electrophoresis and High Sensitivity DNA assay with the 2100 Bioanalyzer System (Agilent Technologies). Library peak size was in the range of 300 to 400 nucleotides. Library concentrations were quantified by fluorometry with the Qubit dsDNA HS Assay system (Life Technologies). For each library, the total DNA amount was required to exceed 1100 ng to be sufficient for three different enrichment procedures.

### Pre-capture sample pooling

We designed two pools of libraries “A” and “B” (Fig. 1), each comprising 12 different libraries. Three were prepared from the NA12891 reference sample, one of them was prepared from our in-lab reference sample E701, and eight were prepared from the DNA samples collected from patients.

Both pools “A” and “B” underwent three independent procedures:

1. Exome enrichment by “RSMU_exome” protocol with the Agilent SureSelect All Exon v6 probe; OR
2. Exome enrichment by “RSMU_exome” protocol with the MGIEasy Exome Capture V4 Probe Set; OR
3. Exome enrichment by MGIEasy protocol with the MGIEasy Exome Capture V4 Probe Set.

For the MGIEasy enrichment protocol (procedure 3), the pools were reduced to eight libraries per pool according to the manufacturer's recommendations^19^. As 8-plex hybridization requires 250 ng of each library, the total DNA amount in a pool was 2 µg.

Following the “RSMU_exome” enrichment protocol (see Supplementary File S1), we pooled 400 ng of each of 12 libraries, so the total DNA amount in the pool was 4.8 μg. The library pools were completely dried using the SpeedVac concentrator (ThermoFisher) at 50 °C.

### Enrichment methods

#### The “RSMU_exome” protocol

*Hybridization.* Dried pools A2, B2 and A3, B3 were combined with 11 μL Cot-1 DNA (1 μg/μL, ThermoFisher) and two adaptor-blocking oligonucleotides with LNA modifications (500 pmol each) (Table 1). Samples were transferred to PCR tubes and denatured at 95 °C for 5 min, followed by a second infinite hold at 65 °C.

**Table 1:** Blocking oligo sequences.

Blocking oligo ID	Sequence 5ʹ–3ʹ (“ + ” = LNA modification, “I” = 2ʹ-deoxyinosine)
Ad-block-1	GAACGACA + TGGC + TACGA + TCCGAC + TT
Ad-block-2	TGTGAGCC + AAGG + AGTTGiiiiiiiiiiTTGTCTTCCT + AAG + ACCGCTTGGCCTCCG + ACTT

After that, we added 14 μL of hybridization buffer thoroughly vortexed and preheated at 65 °C for 10 min to the samples to dissolve any precipitates (the components are listed in Table 2).

**Table 2:** Components in hybridization buffer.

Component of hybridization buffer	Volume per sample, μL
Hyb 1 (20 × SSPE)	9
Hyb 2 (0,5 M EDTA)	0.5
Hyb 3 (50 × Denhardt’s solution)	3.5
Hyb 4 (10% SDS)	0.5
Total volume/sample	13.5

Finally, the mixture of RNA baits (4 μL of the Agilent v6 baits or 6 μL of the MGI v4 baits) with 1 μL of the SUPERase-In (20 U/μL, Invitrogen) blocker preheated at 65 °C for 5 min was added to the samples right in a thermocycler. After slowly pipetting the samples, we added mineral oil to prevent their evaporation. The hybridization mixture was incubated in the thermocycler for 24 h at 65 °C with the lid heated to 105 °C.

*Washing.* Per a single hybridization reaction, 30 μl of C1 streptavidin Dynabeads (10 mg/mL, Invitrogen) were washed three times with 200 μL binding buffer (1 M NaCl, 10 mM Tris–HCl (pH 7.5), 1 mM EDTA) on a magnetic rack and then resuspended in 70 μL of a binding buffer in the LoBind tubes (Eppendorf). After that, Dynabeads were incubated with 3 μg of salmon sperm DNA per reaction for 15 min at a room temperature on a rotator. Prior to the capture, we preheated Dynabeads at 65 °C for 5 min.

The enriched pools were bound to streptavidin Dynabeads and left in a thermal shaker at 65 °C for 30 min. Then, they were washed for three times: we collected the beads on a magnetic rack, removed the supernatant, added 500 μL of prewarmed (for 45 min at 65 °C) wash buffer (0.02X SSC/0.01% SDS), and incubated the samples in a thermal shaker at 65 °C for 10 min. After washing, the samples were dried for 3–4 min on a magnetic stand and resuspended in 31 μL of mQ in new PCR tubes.

Prior to amplification, we denatured the enriched DNA libraries from the Dynabeads by heating samples at 95 °C for 5 min and rapidly collecting the supernatant using a magnetic rack into the new PCR tube.

*Amplification.* The post capture PCR set-up was performed as follows: ½ volume of enriched pool e.g. 15 μl, 8 μl 0.3 µM MGI primer mix (Table 3), 1.5 µL 10 mM dNTP Mix, 10 µL of KAPA HiFi Fidelity buffer (5X) and 1 µL Kapa HiFi HotStart Polymerase (1 U/µL Kapa Biosystems) in a total volume of 50 µL. We used the following PCR program: 3 min at 95 °C, 7 cycles × (20 s at 98 °C, 15 s at 60 °C, 30 s at 72 °C), 10 min at 72 °C. When MGI v4 baits were used for pools 3A, 3B, 10 cycles of PCR were completed for amplification.

**Table 3:** Primer sequences.

Primer ID	Sequence 5–3ʹ
MGIAd_PCR_1	/5Phos/GAACGACATGGCTACGA
MGIAd_PCR_2	TGTGAGCCAAGGAGTTG

Immediately after PCR, the quality and size distribution of the enriched pools were checked by 2% gel electrophoresis, and the entire volume of the PCR product was purified using 1 × SPRI beads (Ampure XP) and eluted in 38 μL of mQ water. Next, the concentration was measured using the Qubit dsDNA HS Assay system (Life Technologies).

### MGIEasy Exome Capture

3A and 3B pools were hybridized and captured with the MGIEasy Exome Capture V4 Probe following the manufacturer’s protocols. The hybridization was performed at 65 °C for 24 h, then the library pools were captured using the streptavidin-conjugated magnetic beads MyOne T1 Dynabeads at room temperature with the subsequent series of washes. After that, the post-capture amplification was performed with 13 cycles of PCR.

### Sequencing

Finally, six pools of the enriched DNA libraries were circularized to generate single-stranded DNA circles. All samples were processed for DNB generation and massive sequencing in the paired-end 2 × 150 bp mode according to the MGI protocol. We loaded one pool per lane into the patterned flowcells in two different runs on the MGISEQ-2000 platform.

### Bioinformatic pipeline

**Figure 2:**
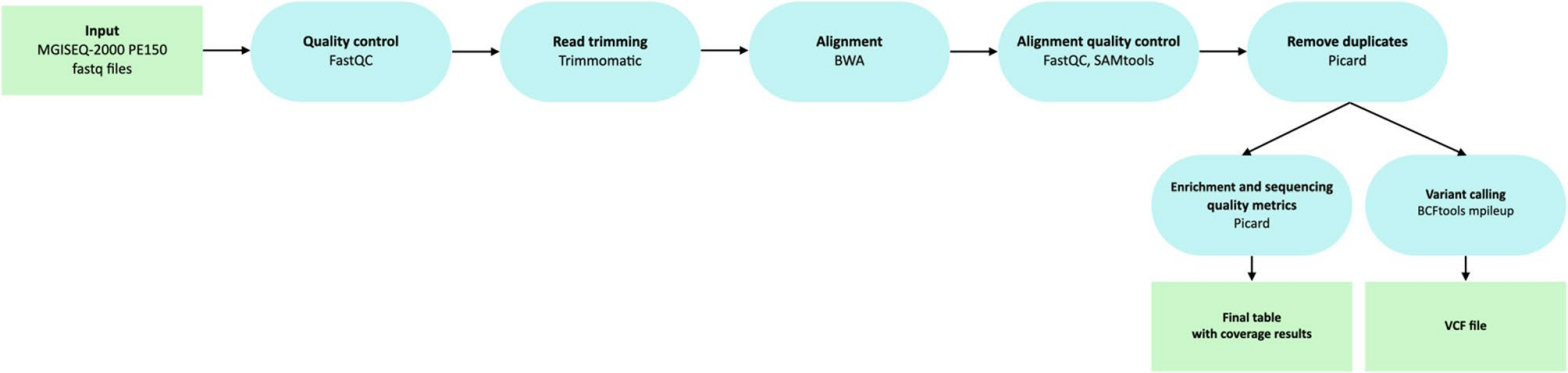
Schematic of the bioinformatic pjpeline for exome data processing. The figure shows the software used for the analysis of the data obtained both during the experiment and downloaded from the open source.

The data processing scheme is shown in Fig. 2. The quality of the obtained fastq files was analysed using FastQC v0.11.9^20^. FastQC results were combined in a single report using multiqQC^21^. Based on the quality metrics, the fastq files were trimmed using Trimmomatic v0.39^22^. Reads were aligned to the indexed reference genome GRCh37 using bwa-mem^23^. SAM files were converted into BAM files and sorted using SAMtools v1.9 to check the percentage of the aligned reads^24^. Based on the obtained bam files, the quality metrics of exome enrichment and sequencing were calculated using Picard v2.22.4^25^ and the number of duplicates was calculated using Picard MarkDuplicates v2.22.4.

The obtained bam files were analyzed following two strategies: the calculation of quality metrics of exome sequencing enrichment and calculation of statistical parameters of the enrichment using NA12891 as a gold standard.

For correct estimation of enrichment and sequencing quality, the NA12891 samples were downsampled to 50 million reads using Picard DownsampleSam v2.22.4. The duplicates were removed from the downsampled samples by Picard MarkDuplicates v2.22.4, and the quality statistics of the obtained data was calculated using Picard CollectHsMetrics v2.22.4 To correctly compare the two enrichment reagent kits, we performed the quality control analysis with the following bed files: MGIEasy v4, Agilent v6, the intersection of MGIEasy v4 and Agilent v6, and all protein-coding regions according to Ensembl database.

The next step to assess the quality of the obtained data was to analyze the quality of SNV and indel calling. Raw reads were aligned using the method described above. In the case of SNV and indel analysis, first, we removed duplicates and then downsampled BAM files to 50 million reads. Then for all samples from each pool, variant calling was performed using bcftools mpileup v1.9, and for all vcf files intersection over union (IoU) values were calculated.

## Results

### Comparison of enrichment methods

Here we show the enrichment protocol for different probes we use in our lab for MGISEQ-2000 sequencing. We enriched the same library pools using two different protocols (our protocol and the protocol from MGI Tech) and showed that our protocol is compatible both with the MGI Tech and Agilent probes.

For enrichment, we suggest pooling 12 libraries (see results for the libraries in detail in Supplementary Table S1, Supplementary Fig. S1) each containing 300–500 ng of DNA. The protocol from MGI recommends pooling no more than eight libraries each containing 250 ng of DNA. The maximum input amount of DNA libraries is 5 µg or 2 µg according to our protocol “RSMU_exome” or the MGI protocol, respectively. Before capture, we block Dynabeads with salmon sperm DNA as Dynabeads are known to bind DNA themselves (1 mg of Dynabeads MyOne Streptavidin C1 typically binds ~ 20 µg of ds-DNA and ~ 500 pmol of ss-oligonucleotides)^26^. In this way, we prevent DNA-RNA hybrid immobilization directly on the Dynabead surface. The samples are washed only in one buffer and under a constant temperature of 65 °C. Prior to the post-capture PCR, we denature the pool with Dynabeads following our experience (see Supplementary Table S2), PCR efficiency in the presence of Dynabeads drops by ~ 25%. We also use the highly processive KAPA Hi-Fi polymerase which had proved itself as the most efficient solution for library amplification in our laboratory.

Although we use only the half of the reaction volume and a fewer number of cycles (7–10 cycles) in the post-capture PCR compared to the MGI Tech protocol (13 cycles), our protocol (Table 4) provides the higher yield of enriched library pools (in ng) indicating it is a more efficient procedure of enrichment and amplification.

**Table 4:** The yield (ng) of enriched DNA library pools for different protocols.

Pool ID	Protocol	Baits	input DNA lib, ng	PCR kit	Cycles post-PCR	output DNA lib, ng
1A	RSMU_exome	Agilent v6	4800	KAPA	7 (½ volume)	139
1B	RSMU_exome	Agilent v6	4800	KAPA	7 (½ volume)	144
2A	RSMU_exome	MGI v4	4800	KAPA	10 (½ volume)	520
2B	RSMU_exome	MGI v4	4800	KAPA	10 (½ volume)	585
3A	MGI	MGI v4	2000	MGI	13	127
3B	MGI	MGI v4	2000	MGI	13	123

### Comparison of probe designs

Each manufacturer of exome enrichment kits strives to reach the most optimal probe design for the most relevant human exome regions in the targeted capture. MGIEasy v4 and Agilent v6 probe design share much similarity (Table 5). For target DNA hybridization, both manufacturers use biotinylated cRNA probes, the MGIEasy v4 probes being 30 bases shorter than the Agilent v6 probes. We measured probe concentration using Qubit RNA HS Assay system (Life Technologies) and established that the Agilent v6 probe concentration is two times higher compared to the MGIEasy v4 probes.

**Table 5:** Exome capture bait designs.

	MGI v4	Agilent v6
Bait type	Biotinylated cRNA baits	Biotinylated cRNA baits
Bait length range, bases	90	120
Bait concentration, ng/μl	114	218
Target size, Mb	59	60
Number of target regions	198 025	243 872
Median Region Size, bp	210	210
.bed file, link	MGIEasy Exome Capture V4 Probe Set, https://en.mgi-tech.com/products/reagents_info/id/9	S07604514_Covered.bed, https://earray.chem.agilent.com/suredesign/index.htm, SureSelect Human All Exon V6 r2, design identification number S07604514

**Figure 3:**
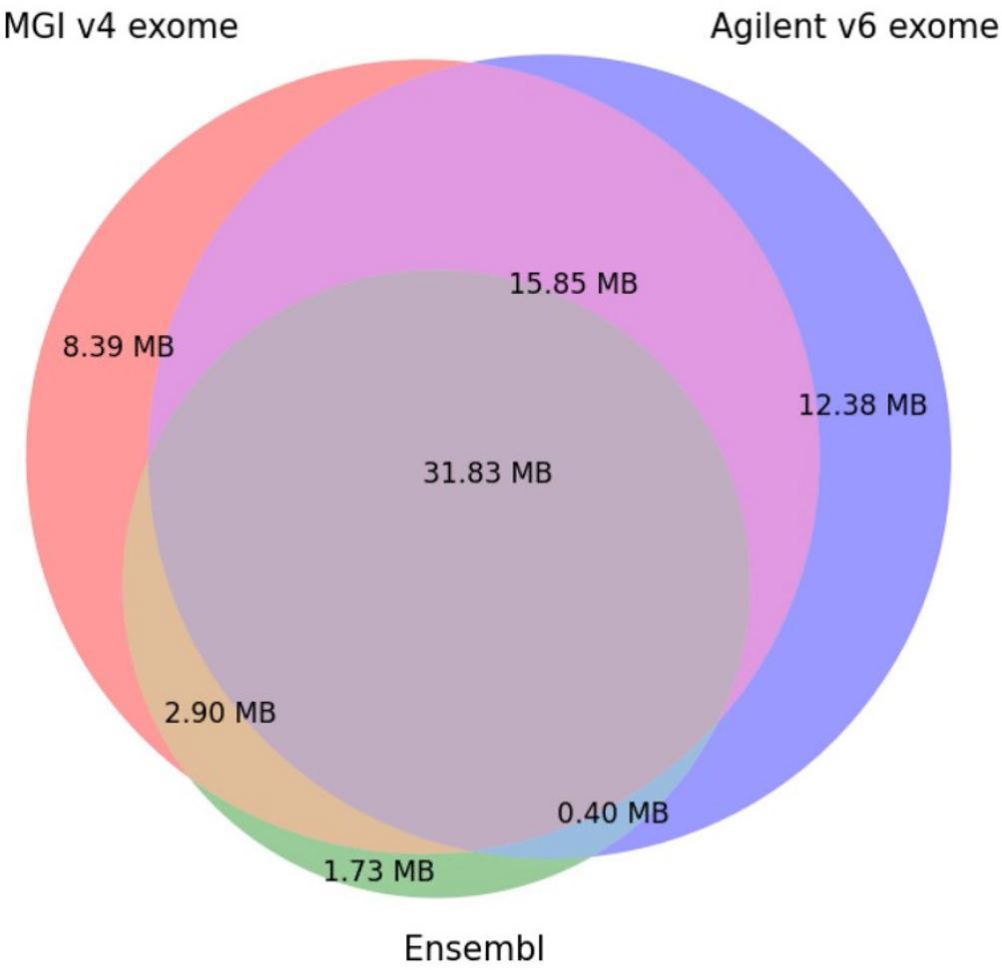
Venn diagram for intersection of MGI v4 exome, Agilent v6 exome, Ensembl coding exons.

The MGIEasy v4 set targets 198 025 regions of the human genome with the size of 59 MB, whereas the Agilent v6 set targets a slightly larger number of regions, 243 872 regions with the size of 60 MB. Median Region Size (bp) is equal to 210 bp in both platforms. We compared bed files from both MGIEasy v4 and Agilent v6 to determine the degree of the intersection between target regions for these two probe sets. We determined the range of target regions using the Ensembl database of protein-coding exons (Ensembl bed file for GRCh37/hg19 assembly, Ensembl genes track for coding exons, obtained from https://genome.ucsc.edu/cgi-bin/hgTables). We visualised the overlapping target regions for the probes of the MGI v4 exome, Agilent v6 exome, and Ensembl coding exons as Venn diagrams with indicated target sizes using matplotlib-venn library (https://github.com/konstantint/matplotlib-venn) (Fig. 3).

Figure 3 shows the overlap areas of all three bed files with regard to their target sizes in megabases.

The percentage of unique target regions for the MGI v4 exome is 11.42% (8.39 MB), for the Agilent v6 exome is 16.84% (12.38 MB) and for Ensembl coding exons it is 2.36% (1.73 MB). The overlap area of all three bed files is 43.32% (31.83 Mb). The overlap area of the Agilent v6 and MGI v4 exomes is 64.89% (47 Mb). The overlap area between MGI v4 and Ensembl (47.26%) is larger than between Agilent v6 and Ensembl (43.86%). The percent of uncovered Enselmbl coding regions by either set is 2.36%.

### Raw data and Pooling balance

For each pool, about 324.5–434.5 million (M) of aligned 150-bp paired-end reads were generated in two MGISEQ-2000 runs.

The results of the fastQC quality check are presented as a single report collected by multiqQC^21^, as well as separate reports for each sample (see Supplementary File S2). Most data are Phred + 35 (average quality per read) which indicates high sequence quality. Therefore, the quality of the sequencing raw data is sufficient for further analysis. In FastQC reports, we often observe slight base imbalance in the “Per base sequence content” parameter at the read start and very rarely at the read ends. Typically, this arises from non-random fragmentation and treatment of DNA ends during library preparation. We estimated the imbalance ratio and trimmed several bases (usually 1–3 bases at the read start and 0–2 at its end) if necessary.

In case of the samples prepared with the Agilent probes, the distribution of Per sequence GC content in fastQC reports has a bimodal structure. Interestingly, GC content distribution across exons in the human genome is bimodal as well^27,28^; therefore, from this point of view, the solution offered by Agilent seems to be a good approximation for our regions of interest.

**Figure 4:**
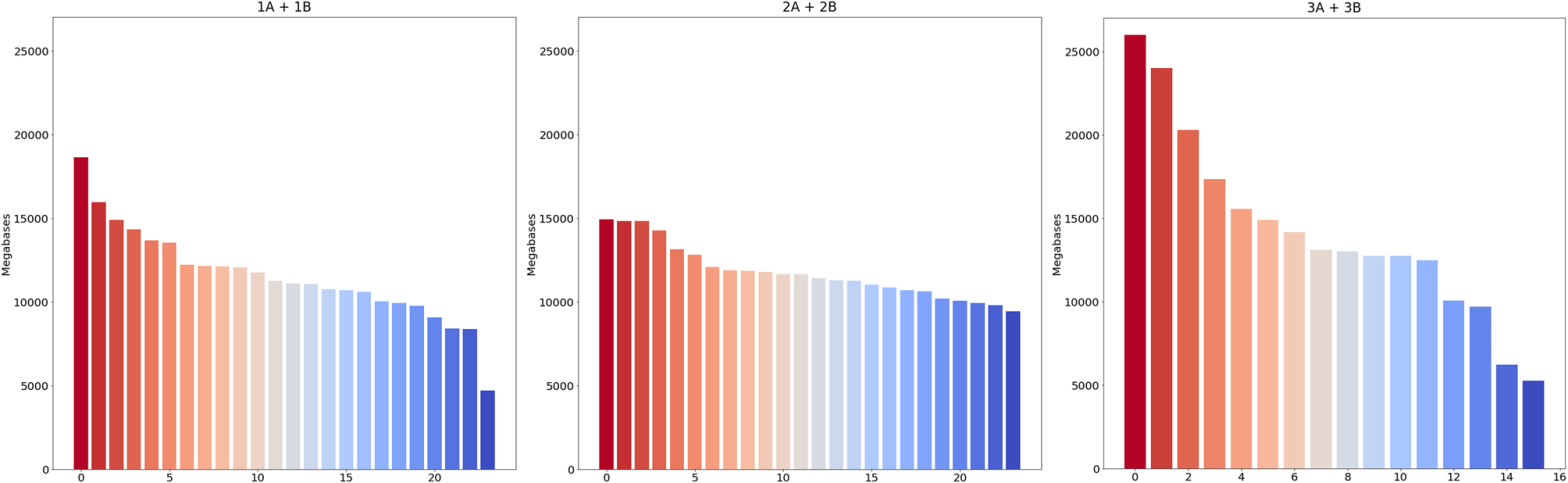
The stacked barplot of the sample sizes across the pools (in Megabases).

**Figure 5:**
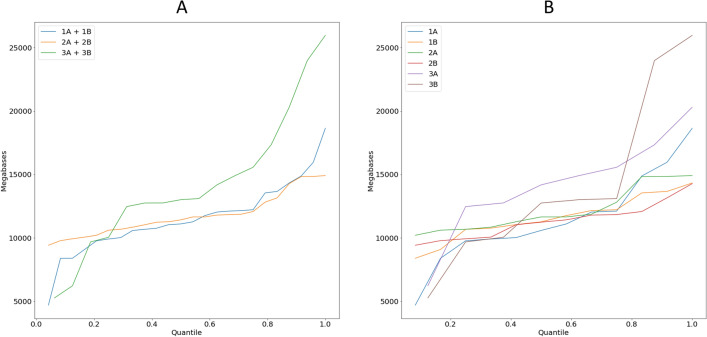
(**A**) The quantile function for combined pools 1A + 2A, 1B + 2B, 3A + 3B. (**B**) The quantile function for each pool.

We obtained an average 65–75 M reads per sample, but within pools for different protocols the variance in the number of reads per sample was: pools of 12 samples 1A–Δ53 M reads, 1B–Δ35 M reads, 2A–Δ22 M reads, 2B–Δ23 M reads; pools of 8 samples 3A–Δ64 M reads, 3B–Δ101 M reads. Figure 4 shows a stacked barplot demonstrating the distribution over the samples in the pools. To illustrate this point better, we show the diagrams of quantile function (Fig. 5) which clearly demonstrate the dynamics of data size distribution. As can be seen, the MGI protocol and MGI v4 exome probes denoted by the green line (pools 3A + 3B), are more imbalanced compared to the other pools (Fig. 5A). The sharp jump at the beginning of the quantile function, indicates that the proportion of samples receiving less than 12 000 megabases is 0.4. Figure 5B shows the imbalance between pools 3A and 3B in more detail. Such specificity of data obtaining does not allow achieving an even distribution of MB over the samples, potentially leading to under-coverage of some regions in the samples in the pool. Although pools 1A + 1B show slight imbalance at the very beginning, the balance of the obtained sample data is higher for the RSMU_exome protocol than for MGIEasy protocol.

### Enrichment quality

At least 99% of reads per sample were mapped on human DNA with BWA. Using Picard, for each sample in a pool, we calculated the standard metrics: on-target %, off-target, mean coverage, covered × 5, × 10, × 20 values etc. (see Supplementary Table S3). For pools 1A and 1B, we obtained 6.3–15.4% (mean 8.11%) of duplicates, for 2A and 2B, 15.2–22.5% (mean 18.375%), for 3A and 3B, 10–26.5% (mean 19.78%). The higher the number of reads, the higher the number of duplicates. The minimum value for the Covered × 10 parameter for the samples with the lowest read number were 97.61%, 97.21%, and 89.62% for approaches 1, 2, and 3, respectively. The lowest mean coverage of samples in pools 1A and 1B was 69.1 (52.39 M reads) and 67.73 (52.37 M reads), in pools 2A and 2B = 79.82 (58.03 M reads) and 74.26 (54.48 M reads), in pools 3A and 3B = 43.81 (35.92 M reads) and 30.9 (31.57 M reads) respectively.

### Comparison of methods using downsampled Platinum Genomes

#### Mapping: on-target percentage and duplication rate

**Figure 6:**
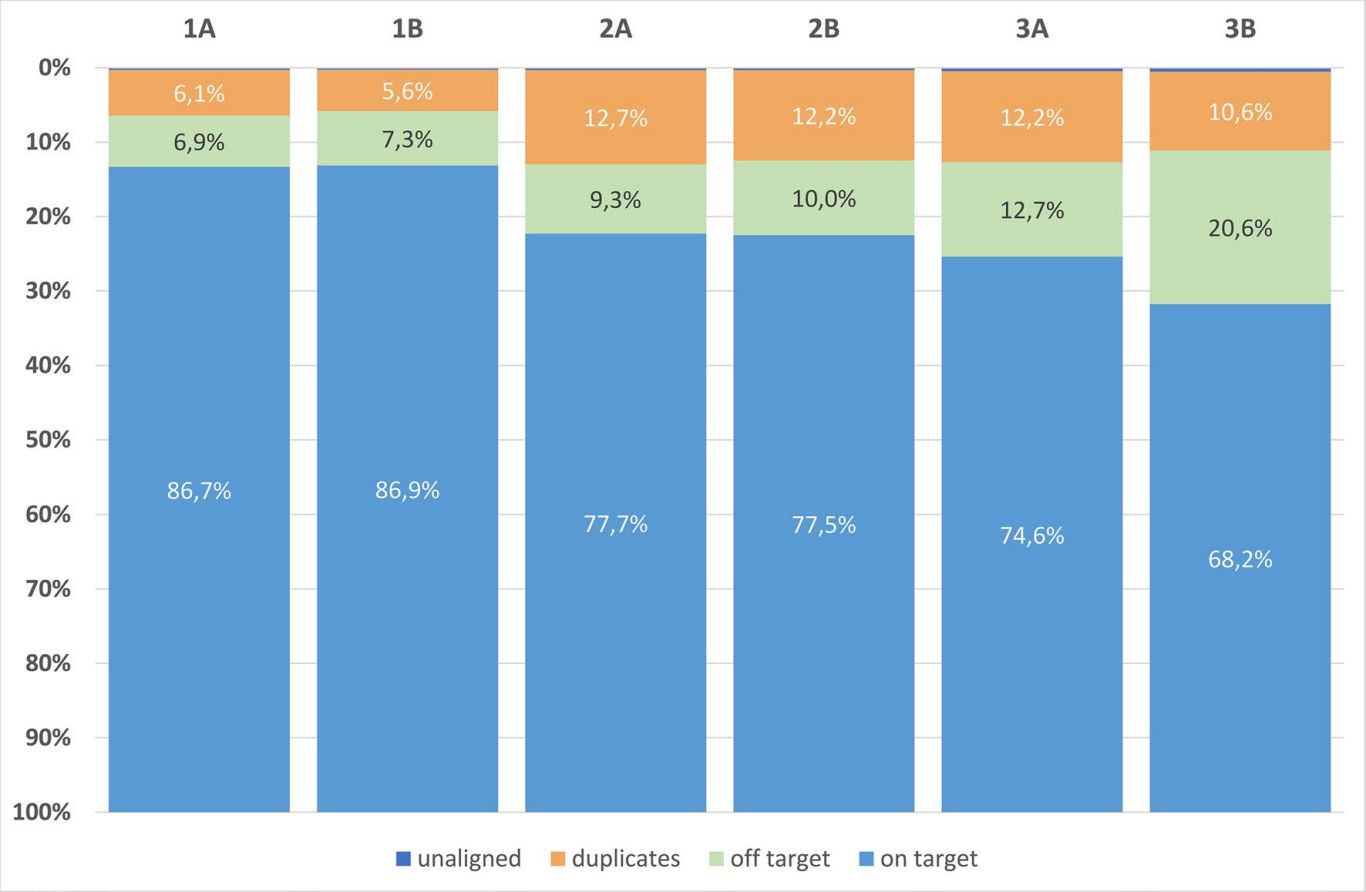
The stacked barplots for averaged on-target, off-target, duplicates, and un-aligned reads values in the pools (for downsampled NA12891 results).

To compare three different protocols correctly, we performed downsampling (subsampling of paired-end reads) of raw data to 50 million reads for the samples from the Platinum Genomes using Picard v2.22.4. After that, we estimated the percentages of on-target, off-target, and un-aligned reads and duplication percentage for each pool (Fig. 6). In case of the first approach (the "RSMU_exome" protocol with the Agilent v6 probes), the duplicate number was the lowest (5.9%) while it was about 11.9% in case of the second or third approaches (the RSMU_exome and MGIeasy protocols with the MGI v4 probes). For all approaches, the number of un-aligned reads did not exceed 1%. The mean percentage of mapped off-target reads was 7.1% for approach 1 (RSMU_exome protocol + Agilent v6), 9.7% for approach 2 (RSMU_exome protocol + MGI v4), and 16.7% for approach 3 (MGIeasy protocol + MGI v4).

### Coverage analysis

We estimated enrichment efficiency by comparing the coverage depth of target regions for three approaches. We used only NA12891 samples downsampled to 50 M and, if necessary, to 40 M, 30 M, and 20 M reads in the pool coverage comparison. Coverage values for the samples in pools A and B were averaged as we did not detect any significant differences between the results for pools A and B for each approach which indicates a high level of technical reproducibility. Coverage was evaluated based on three bed files: bed file corresponding to the probes used for sample preparation, bed file for Ensembl coding exons, and bed file of shared regions for MGIEasy v4 и Agilent v6 (cross bed). See Supplementary Table S4 for Picard parameters for all NA12891 samples downsampled to 20 M, 30 M, 40 M, and 50 M reads.

**Figure 7:**
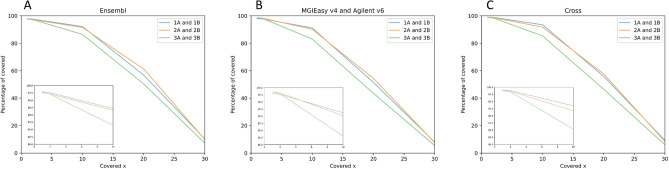
Dependence of region coverage quality for different depths on 50 Mb read samples for regions corresponding to bed files: (**A**) Ensembl, (**B**)-sample probes (MGI v4 or Agilent v6), (**C**)-intersection regions of bed files MGI v4 and Agilent v6.

The fraction of target regions covered at least one time for the compared approaches differed slightly on downsampled to 50 M samples (Fig. 7) with the MGI probes averaging almost the same for both our protocol (98.60%) and the original MGIeasy protocol (98.59%); for Agilent v6 probes the value was 97.93%. At the same time, the trend changes markedly for “on-target covered × 10”, where our approach RSMU_exome with Agilent v6 probes maintains coverage at a high level, while the MGIeasy protocol loses dramatically in values. Thus, the average “on-target covered × 10” were: 95.81% (min = 94.25%) for RSMU_exome protocol + Agilent v6; 94.96% (min = 94.4%) for RSMU_exome protocol + MGI v4; 91.13% (min = 88.15%) for MGIeasy protocol + MGI v4. The parameter “on-target covered × 30” is on average > 75% for the RSMU_exome, and lower by 12% for the MGIeasy (on average > 63%).

Figure 7 shows the performance of exome protocol in terms of coverage quality on normalized reference samples.

For bed Ensembl coding exons (target size = 35.4 Mb) and for overlapped target regions MGI v4 and Agilent v6 (cross bed, target size = 47.9 Mb) the trend remained, the RSMU_exome approach on both bed files was better.

**Figure 8:**
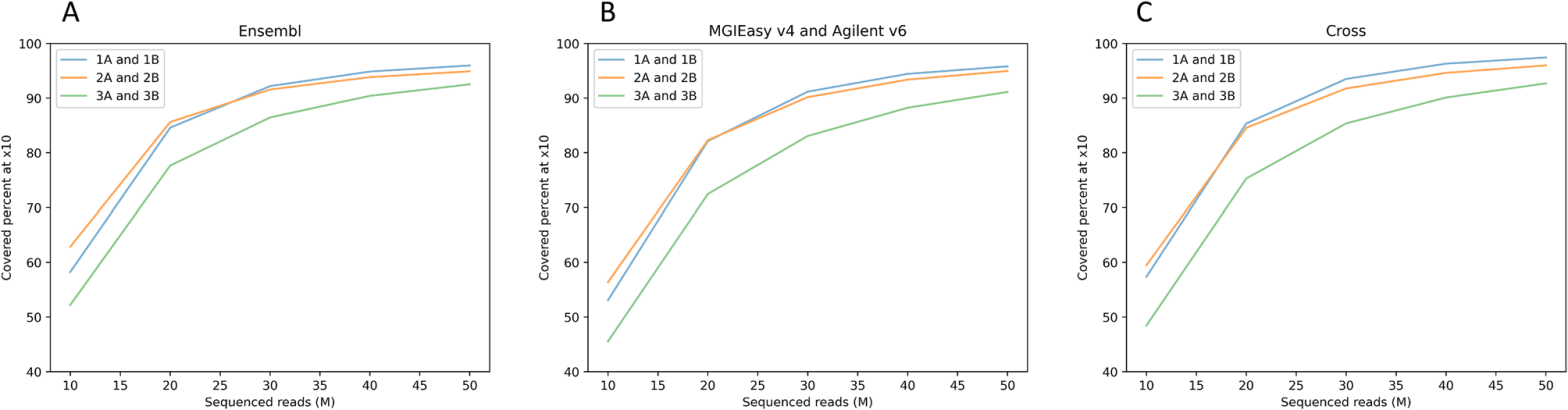
The percentage of regions with × 10 coverage for downsampled samples and corresponding bed files: (**A**) Ensembl, (**B**) sample probes (MGI v4 or Agilent v6), (**C**) overlapping regions of bed files MGI v4 and Agilent v6.

Furthermore, we studied the percentage of on-target covered × 10 for the samples downsampled to 10 M, 20 M, 30 M, 40 M, 50 M reads (Fig. 8). The RSMU_exome protocol shows the best result for all bed files: on average, even 30 M reads are sufficient for × 10 coverage of more than 90% of the on-target regions for Agilent v6, MGI v4, and the cross bed, and more than 91% for Ensembl coding regions. For the MGIEasy protocol, comparable results were obtained from 50 M reads per sample. At the same time, the curves reached the plateau with the increment of ~ 2% on-target covered at ≥ 10 × for the RSMU_exome protocol and both Agilent v6 and the MGI v4 probes at 40 M reads in contrast to the MGIeasy protocol for the MGI v4 probes which indicates a higher hybridization and capture efficiency for our protocol. Thus, fewer number of reads are required to obtain the same completeness of exome coverage when the RSMU_exome protocol is used.

 Figure 8 shows the performance of exome protocol in terms of coverage quality and sufficient sequencing depths.

### GC content

**Figure 9:**
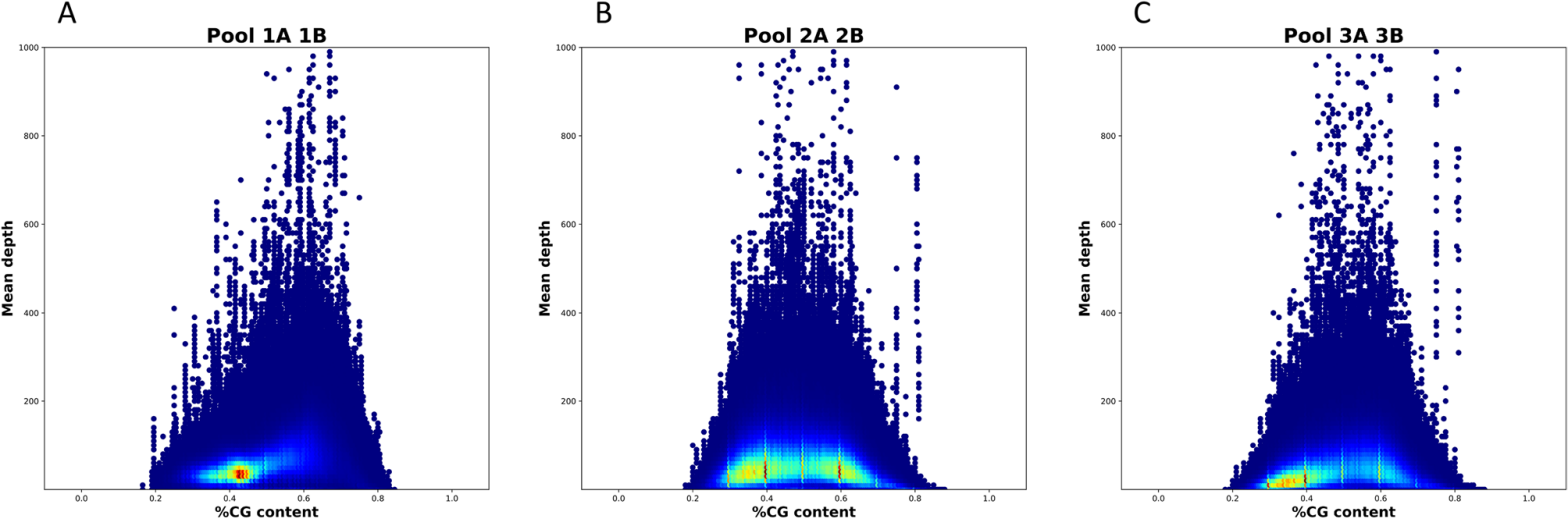
Density plot of %GC Content vs Mean Depth for: (**A**) RSMU_exome + Agilent v6; (**B**) RSMU_exome + MGI v4; (**C**) MGIeasy capture + MGI v4. Here we show a 2D density plot of %GC Content vs Mean Depth parameters calculated by Picard HsMetrics v2.22.4. We obtained the data for this plot by merging all samples from the corresponding pools (1A + 1B, 2A + 2B, 3A + 3B) with Picard 2.22.4 . Density estimation was performed using 2D histograms. More specifically, we chose data points in a fixed rectangle (%GC Content ∈ (0;1) and Mean Depth ∈ (0;1000) ), then we split this rectangle into an evenly spaced grid of the size 200 × 100, and counted the number of data points in each cell of the grid. Finally, we normalized the grid to the (0,1) range and plotted it using "jet" colormap from the matplotlib library (https://matplotlib.org/).

Most enrichment techniques show fall read depth in GC-rich and GC-poor regions. We compared the pools based on this parameter using a density plot (Fig. 9). As expected, all three approaches showed a drop in the coverage depth in the regions with extremely low (< 20%) or high (< 80%) GC content. Interestingly, the pools 2A + 2B и 3A + 3B enriched with the MGI v4 probes following different protocols show a more uniform coverage upon GC content between 40 and 60%, in contrast to the pools 1A + 1B enriched with the Agilent v6 probes which have a peak of over covered (> × 400) target regions shifted to the 50% and 70% GC content. For the pools 1A + 1B, the maximum coverage density lies in the range of 40%-50% GC. The difference between the pools 2A + 2B and 3A + 3B (MGI v4 probes) is insignificant, however, the coverage density between 40 and 60% GC is more uniform for the pools 2A + 2B prepared following the RSMU_exome protocols.

### SNV and INDEL analysis

For SNV analysis, we used the data filtered following the algorithm described above including de-duplication and downsampling to 50 million reads per sample. Further, we obtain information about gene sequence variations using bcftools mpileup v1.9. We filtered them based on the coverage leaving only those variants covered by at least 13 × and those localized in target regions.

**Figure 10:**
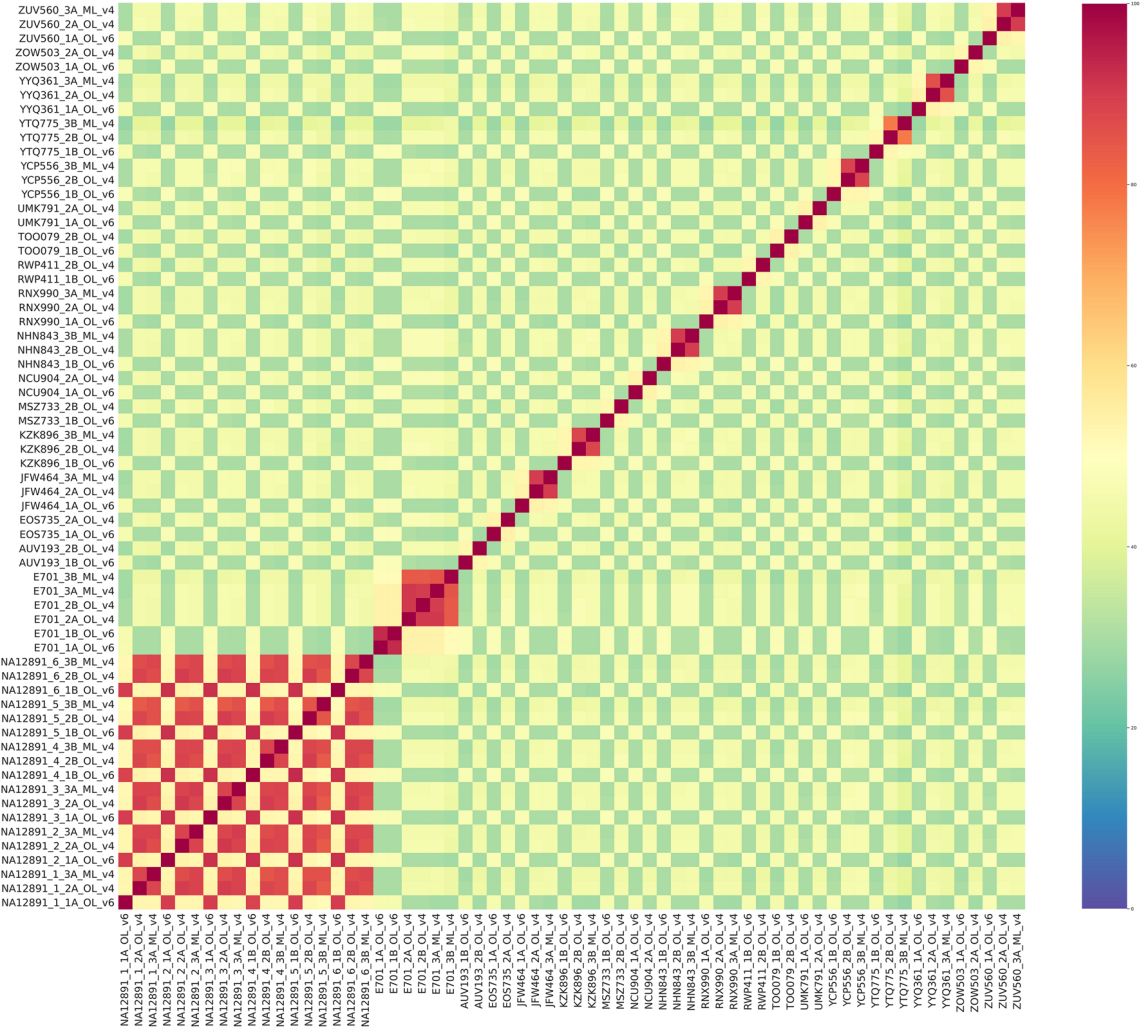
SNV Heatmap for all experimental samples. Visualisation of IoU SNV results for all samples filtered by target regions.

To assess the similarity of the obtained data, we calculated the IoU values for all samples from the six pools. Figure 10 shows two clearly distinguishable clusters formed by samples NA12891 and E701. These clusters provide evidence that the data obtained for the sample NA12891 represent replicate libraries although they were obtained using different protocols. Meanwhile, for replicates of NA12891 samples from pools A and B within each approach, the IoU values show maximum results, which shows high reproducibility of results for different pools for the same approach.

**Figure 11:**
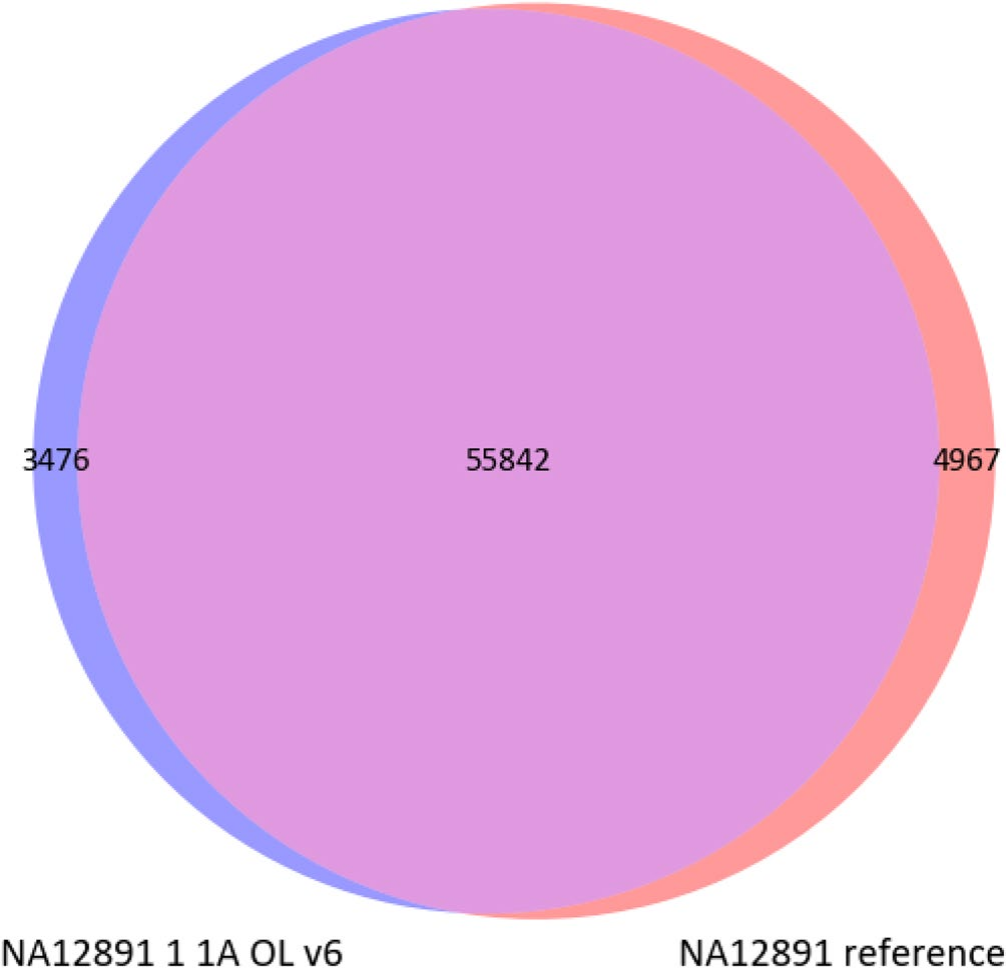
Venn diagram for the intersection of SNV and indel for a randomly chosen sample NA12891 from the pool 1A and the reference genome of sample NA12891. The data were filtered for depth of coverage over 13 reads. The 55 715 SVNs and indels found were a complete match to the reference genome by genotype.

To determine if the results for the Platinum Genome are accurate, we used the genome sequencing data for the NA12891 sample from an open source (ftp://ftp.sra.ebi.ac.uk/vol1/fastq/SRR622/SRR622458). Based on IoU, the position, the substitution (SNV or indel), and genotype, we evaluated how calling results for one of our samples NA12891 fits into the reference genome analysed following our bioinformatic pipeline. We used a Venn diagram to visualize this, Fig. 11 shows that for the samples filtered by the Agilent v6 bed file, our sample NA12891 from the pool 1A is almost completely fits into the reference genome NA12891_ref37 filtered by the target regions of the Agilent v6 exome with the coverage depth cutoff of 13 reads. This Venn diagram implies that the results from our data are correct, and bcftools mpileup v1.9 allows correct variant calling with high accuracy.

**Figure 12:**
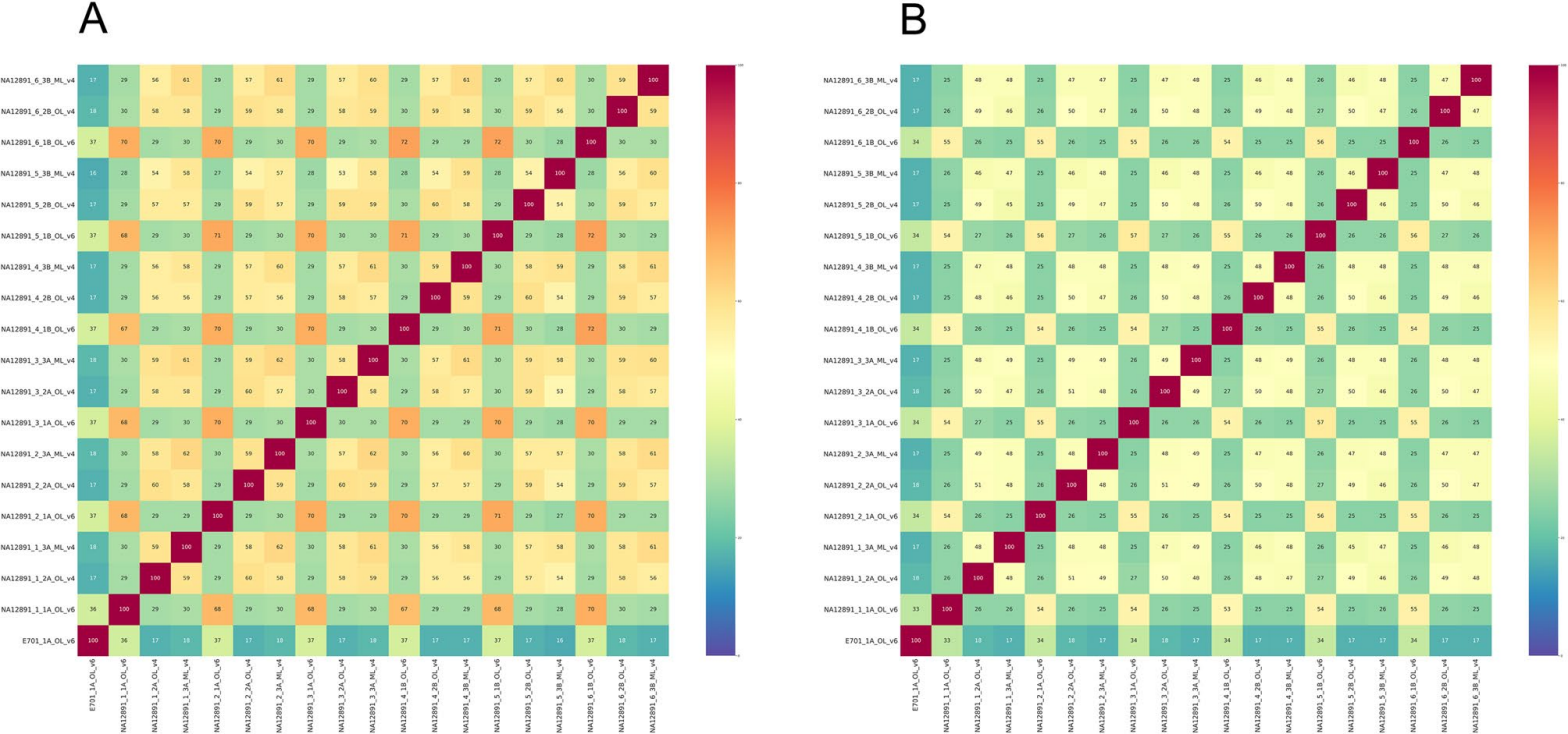
IoU heatmaps for indel from the sample NA12891 and one random sample E701 from the pool 1A. Visualisation of the IoU results for insertions (**A**) and deletions (**B**), that can be called using bcftools mpileup v1.9.

We used the IoU metrics to estimate the quality of indel calling on our data pre-filtered by the target regions using bcftools mpileup v1.9. We calculated the IoU values only for the lines from vcf containing insertions and deletions. Based on the results, we constructed two heatmaps (Fig. 12) for insertions and deletions separately. For ease of comprehension, we added a random sample E701 from the pool 1A.

As we can see in the heatmap, indel calling with bcftools mpileup v1.9 allows for the better calling of deletions than of insertions. Samples from different pools have less similarity than samples within pools, and we can see a significant difference between the CNV for sample NA12891 and sample E701.

We collected statistics from the pools for variant calling data. All data from the pools were sorted by their respective bed file and by target regions for Ensembl coding exons. Using this estimation approach, we evaluated the parameters of the number of all detected SNV and indel and of those included only into target regions.

The mean total number of SNVs in on-target regions was 57 189, (RSMU_exome + Agilent v6), 54 094 (RSMU_exome + MGI v4), 54 044 (MGIeasy protocol + MGI v4) when using the 50 M read sets in all samples.

We varied the filtering parameters for the detected variants. The results are shown in Supplementary Table S5. We noticed that the parameters QUAL > 20 and QUAL > 30 provide a significant cut-off across all substitution numbers but almost do not vary in the target regions. The most strict cut-off for the variants with the coverage depth exceeding 13 reads (DP > 13) and a parameter QUAL > 30 insignificantly affected the number of variants. For instance, the SNV number for the pool 1A changed by 3.1% (from 57 189 to 55 407) which is not critical. We also noticed that the higher values of the absolute numbers of all SNV and indel from the full vcf and harboured in the target regions are typical for the pools 1A and 1B. For the most strict SNV cut-off (DP > 13, QUAL > 30), the average variant number in a target is 55,000, 50,000, and 49,000 variants for the pools 1A + 1B, 2A + 2B, and 3A + 3B, respectively. The 1st approach demonstrated the best results. For the same probe set (MGIEasy v4) and different enrichment protocols, our protocol allows obtaining 2% more qualitative calling results from the same sequencing data amount compared to the MGIEasy protocol.

In general, we can conclude that for all cut-offs, average SNV and indel values in the target regions for the samples obtained with Agilent v6 is higher than for the MGI v4. However, we did not detect any significant changes in Ensembl genes track for the coding exons between the pools.

### Statistical analysis

To estimate the quality of SNV and indel detection, we used the data from variant calling in the Platinum Genome for all six pools. All pools were pre-filtered by the target regions for a corresponding set and with the coverage depth exceeding 13 reads (DP > 13) and a parameter QUAL > 20. We used Platinum Genome data as a reference that also were pre-filtered by the target regions (MGI v4, Agilent v6).

To assess SNV and indel detection quality, we employed sensitivity, precision, and F-measure metrics. We chose those metrics, as SNV and indel detection is a task of binary classification into two categories, and these metrics are most commonly used for such tasks.

Sensitivity estimates the probability of a position in a genome identified as an SNV/indel by a variant calling method in truth being SNV/indel.

$$SNV\;Sensitivity = \frac{In\;PG\;SNV}{{In\;PG\;SNV + Not\;in\;PG\;SNV}}$$

$$Indel\;sensitivity = \frac{In\;PG\;indel}{{In\;PG\;indel + Not\;in\;PG\;indel}}$$

Precision estimates the probability of a real SNV/indel being determined as an SNV/indel by a variant calling method.

$$SNV\;Precision = \frac{In\;PG\;SNV}{{In\;PG\;SNV + Not\;in\;PG\;indel}}$$

$$Indel\;Precision = \frac{In\;PG\;indel}{{In\;PG\;indel + Not\;in\;PG\;SNV}}$$

F-measure is a combined metric. It aggregates sensitivity and precision into one metric using harmonic mean. The higher sensitivity and precision, the higher the F-measure.

$$SNV\;F_{measure} = \frac{{2{*}SNV\;Sensitivity{*}SNV\;Precision}}{SNV\;Sensitivity + SNV\;Precision}$$

$$Indel\;F_{measure} = \frac{{2{*}indel\;Sensitivity{*}indel\;Precision}}{indel\;Sensitivity + indel\;Precision}$$

These three metrics provide a good understanding of the method quality.

Additionally, we want to note, that SNV and indel metrics cannot be considered independent since SNV/indel detection is not two separate tasks but one joint task. Mathematically speaking, these metrics are derived from the same confusion matrix.

Thus, we can see that the estimation quality metrics of SNV and indel detection demonstrate excellent results (Tables 6, 7, more detailed results are shown in Supplementary Table S6 and S7). Moreover, the SNV and indel number in a target exonic regions demonstrates slightly better results for the samples from the pools 1A and 1B prepared following our protocol. These results indicated the high quality of the data which allow detection of the maximum number of SNVs and indels.

**Table 6:** Mean results for variation accuracy estimation by comparison with reference Platinum Genome for SNV (PG).

	Protocol	Pool	Total SNV	In PG SNV	Not in PG SNV	PG-specific SNV	Samples-specific SNV	Sensitivity (%)	Precision (%)	F-measure (%)
NA12891 libraries	RSMU_exome	1A + 1B	52,878	52,875	2	2831	1775	99.976	99.995	99.986
	RSMU_exome	2A + 2B	49,251	49,250	1	3733	1652	99.976	99.998	99.987
	MGI	3A + 3B	47,821	47,819	2	5165	1581	99.977	99.996	99.987
Total			49,983	49,981	2	3910	1669	99.977	99.996	99.987

Total SNV-number of bases detected as SNV. In PG SNV-number of bases detected as SNV and being SNV in reference PG. Not in PG SNV-number of bases detected as SNV and being indel in reference PG. PG-specific variations SNV-number of bases that are SNV in reference PG, but not called in our PG at all.

**Table 7:** Mean results for variation accuracy estimation by comparison with reference Platinum Genome for indel (PG).

	Protocol	Pool	Total indel	In PG indel	Not in PG indel	PG-specific indel	Samples-specific indel	Sensitivity (%)	Precision (%)	F-measure (%)
NA12891 libraries	RSMU_exome	1A + 1B	3161	3148	13	650	903	99.927	99.605	99.765
	RSMU_exome	2A + 2B	3110	3098	12	1084	1133	99.963	99.620	99.791
	MGI	3A + 3B	2898	2887	11	1294	1007	99.937	99.627	99.781
Total			3056	3044	12	1009	1014	99.942	99.617	99.779

Total indel-number of bases detected as indel. In PG indel-number of bases detected as indel and being indel in reference PG. Not in PG indel-number of bases detected as indel and being SNV in reference PG. PG-specific variations indel-number of bases that are indel in reference PG, but not called in our PG at all.

## Discussion

To perform high quality exome sequencing analysis of bulk libraries in the laboratory on the MGISEQ-2000 sequencer, we tested the MGIEasy v4 enrichment protocol and being unsatisfied, we designed our own enrichment protocol. We thoroughly studied the known protocols for the common kits for exome enrichment as well as their comparative studies^29–32^, and we focused on high quality enrichment of multiplexed samples with minimal sequencing.

We used the Platinum Genome NA12891 for the protocol validation. To minimize PCR errors and a duplicate number, we decreased the number of post-capture PCR cycles to 7 cycles by introducing the hybridization and capture protocol modifications described above. One of the specific features of the probe preparation for MGISEQ-2000 sequencing is a stage of circularization of the prepared libraries which requires no less than 80–100 ng of libraries. Therefore, we designed an enrichment protocol to obtain the yield sufficient for the further pool processing with the minimum number of PCR cycles.

We increased the maximum amount of libraries in a pool up to 12 which is higher than the amount used in the MGIEasy Exome and Agilent SureSelect protocols (they use no more than 8 samples per a pool) and elevated the amount of the introduced DNA libraries up to 400 ng per pool. It was shown that keeping 500 ng per sample per pool, regardless of the total amount of DNA entering the enrichment, the number of duplicates did not increase and sample coverage remained uniform^33^. We add the Salmon Sperm DNA only in the capture reaction but not in the hybridization reaction as we assumed that it may hybridize with probes targeting conserved regions of the human genome and thus reducing the efficiency of the reaction.

Evidently, pre-capture multiplexing makes the price of enrichment 2.5–4 times lower and reduces the time required for the procedure which is especially important for laboratories with little automation^33–36^. However, obtaining an equal amount of data after multiplexed enrichment is quite complicated. In many works, pooling evenness is described rather vaguely or is not discussed at all, and the differences in the coverage of samples in a pool can reach 10 times^9,34,35,37^. For instance, in^35^, 16 samples were pooled prior to the enrichment following the Agilent SureSelect XT protocol with no decrease in enrichment quality. However, the difference in the number of M read per sample might reach 16 times. In another work, the variation in the coverage of samples in a pool reached 10 times for the Nextera kit (Illumina)^9^. In the work performed in the Center for Inherited Diseases, upon pre-capture pooling following the IDT protocol or the Roche and Twist protocols^34^, the variation in data amount between the samples from the pools reached 5 and 2–3 times, respectively. Therefore, the main disadvantage of pre-capture multiplexing lies in the fact that the samples underrepresented in a pool require not only additional sequencing but also an additional enrichment which makes an analysis more labour intensive and expensive. Following the MGI enrichment protocol, the difference in the amount of data per sample reached × 4 and three samples out of 16 failed to pass the threshold of on-target regions covered by 10 × greater than 95% (samples NA12891_4_3B = 94.73%, YYQ361_3A = 91.46%, YTQ775_3B = 86.57%). These samples have to be additionally enriched. Following the RSMU_exome protocol, the variation in the data amount between the samples was less pronounced with no sample failing to pass the threshold. The lowest value of on-target regions covered × 10 for 48 samples in the pools enriched with both the Agilent and MGI probes was 96.09%, which is a clear advantage of our technique.

After accurately comparing the samples obtained by all protocols with the normalized coverage, we were satisfied with the results obtained following our own RSMU_exome protocol using both Agilent v6 and MGI v4 probes. Both technologies yielded over 80–90% of bases on-target, although for the latter we obtained on average ~ 2 times more duplicates. Routinely used Agilent probes showed slightly better on-target % and coverage of coding exons from the Ensembl database. However, taking into account the other amount of evidence^6–10^, no probes fully covered the sequences of all coding exons. There are certain regions that escape both exome sequencing and genome sequencing when^3,27^ using short reads, in particular, an increased number of genomic repeats or the presence of pseudogenes.

The specific features of sample preparation for exome sequencing can affect the quality of variant calling upon equal target sizes^7^. The quality of calling results with the MGI v4 probes was higher in case of the RSMU_exome protocol compared to the Chinese protocol. Following the RSMU_exome protocol while using the Agilent v6 probes, results of variant calling were comparable to the results of other researchers^3,6,7^.

The uniformity of coverage depth of target regions can be affected by GC-content i.e. low coverage depth may be caused by a high (> 60%) and low (< 40%) GC-content in a target. Such regions affect the coverage by decreased hybridization efficiency (affected by a probe design) and post-capture PCR^38,39^. As expected, all three approaches introduced bias in the regions with extremal GC content, and the RSMU_exome protocol with the MGI v4 probes provided slightly higher uniformity in the regions with the GC-content lying between 40 and 60%. Furthermore, the coverage with regard to GC content obtained with the Agilent SureSelect v6 probes was similar to the Agilent SureSelect QXT kit used in the previous study by García-García et al.^8^.

Currently, there are few reagent kits compatible with MGI Tech sequencing machines and suitable for specific tasks which allow the manufacturers to maintain high pricing. Our modifications of the protocol for library preparation, pooling, library enrichment, and washing proved to be four times cheaper than the solutions provided by MGI Tech (calculated per sample, not including sequencing).

For the first time, we suggest a protocol as an alternative to commercial protocols which surpasses ready-to-use manufacturers' solutions in quality demonstrating a high performance in terms of capture uniformity and on-target coverage with the Agilent v6, and MGI v4 probes. For convenience, we describe the full RSMU_exome protocol in the Supplementary File S1.

## Supplementary Information

Supplementary Information 1.

Supplementary Information 2.

Supplementary Information 3.

Supplementary Information 4.

Supplementary Information 5.

Supplementary Information 6.

Supplementary Information 7.

Supplementary Information 8.

Supplementary Information 9.

Supplementary Information 10.

## Data Availability

Fastq files for each library of NA12891 sample in all 6 pools were deposited in the NCBI open-access sequence read archive (SRA) under BioProject ID PRJNA667840.
