# Supplementary figures and images for: System analysis of the sequencing quality of human whole exome samples on BGI NGS platform
Source: Sci Rep. 2022 Jan 12;12:609. doi: 10.1038/s41598-021-04526-8 (PMC8755732; doi:10.1038/s41598-021-04526-8)

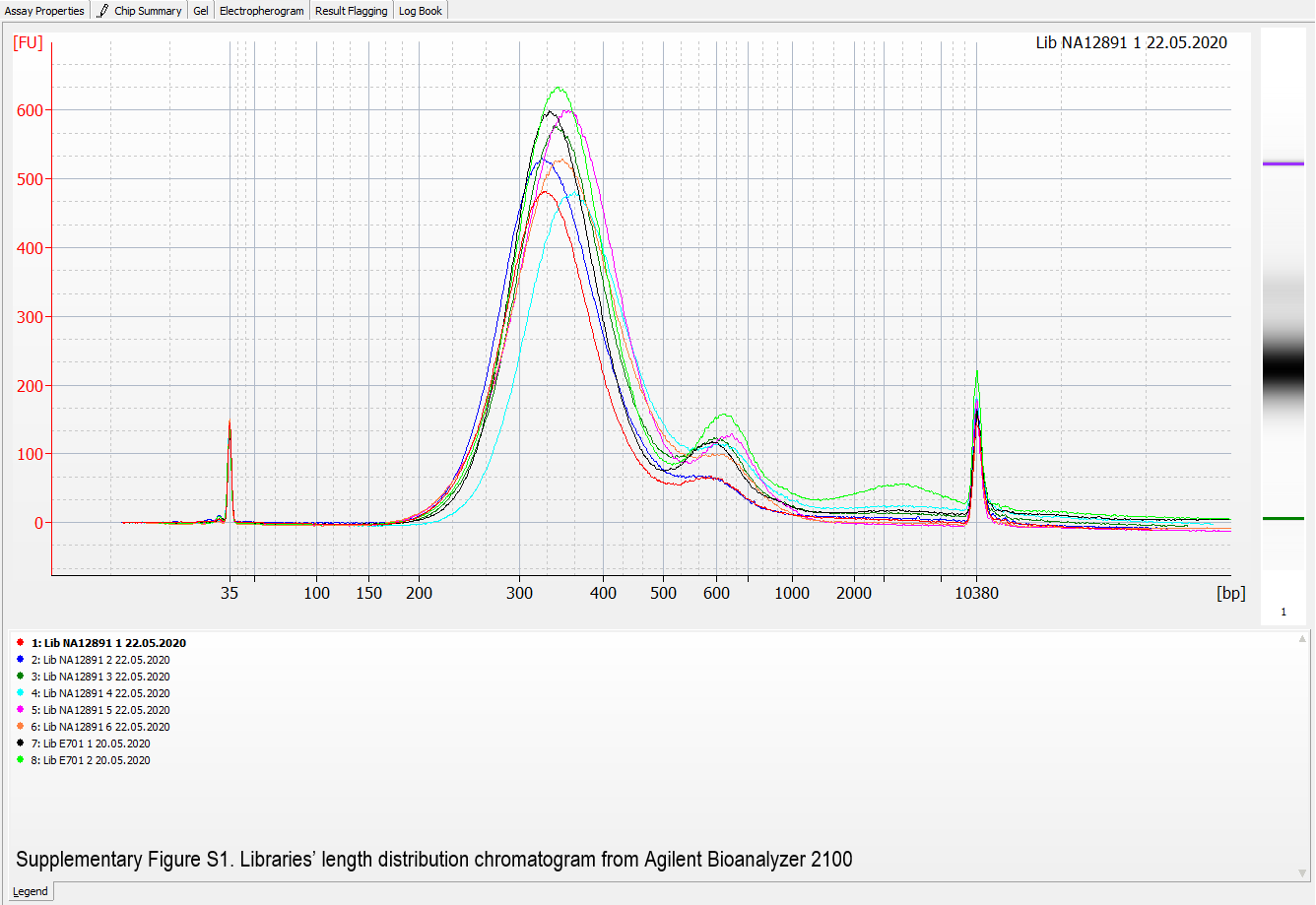

Supplement: Supplementary file 1 — Supplementary Information 1. [file 41598_2021_4526_MOESM1_ESM.png]
